# Safety of locally applied antibiotics in orthopaedic trauma surgery: descriptive results from a prospective cohort study

**DOI:** 10.5194/jbji-11-95-2026

**Published:** 2026-02-10

**Authors:** Niels Vanvelk, Esther M. M. Van Lieshout, Leendert H. T. Nugteren, A. Cornelis Plaisier, Rosalya Van der Pot, Corine Bethlehem, Willem-Jan Metsemakers, William T. Obremskey, Michael H. J. Verhofstad

**Affiliations:** 1 Trauma Research Unit, Department of Surgery, Erasmus MC, University Medical Center Rotterdam, Rotterdam, the Netherlands; 2 Department of Hospital Pharmacy, Erasmus MC, University Medical Center Rotterdam, Rotterdam, the Netherlands; 3 Department of Trauma Surgery, University Hospitals Leuven, Leuven, Belgium; 4 Department of Development and Regeneration, KU Leuven, Leuven, Belgium; 5 Department of Orthopaedic Surgery and Rehabilitation, Vanderbilt University Medical Center, Nashville, TN, USA

## Abstract

Antibiotic therapy holds an integral role in the prevention and treatment of fracture-related infection (FRI). Because local concentrations after systemic administration are limited by the potential for side effects, the local application of antibiotics serves as a valuable adjunct. One concern with the local administration of high amounts of aminoglycosides and vancomycin is the absorption into the systemic circulation, leading to detrimental effects on renal function. In this study, serum antibiotic concentration and renal function were measured in patients treated for open fractures or FRI using local antibiotics at 6, 24 and 48 h postoperatively. Afterwards, laboratory analyses were continued daily until the concentration dropped beneath the lower limit of quantification (LLOQ) (0.22 mg L^−1^ for gentamicin, 0.70 mg L^−1^ for vancomycin). Gentamicin concentration, vancomycin concentration and glomerular filtration rate were measured 272, 60 and 277 times in 52 patients after 82 surgeries, respectively. The LLOQ for gentamicin was exceeded in 14 surgeries (17 %, median concentration 0.3 mg L^−1^). The highest measured antibiotic level was 1.0 mg L^−1^, well below the generally accepted toxic trough level of 2.0 mg L^−1^. Although the total quantity of antibiotics delivered via beads was less than that provided by an antibiotic spacer, the use of beads more frequently yielded measurements exceeding the established threshold. None of the vancomycin measurements surpassed the LLOQ. The results of this study suggest that the current clinical use of locally applied antibiotics in orthopaedic trauma surgery is safe in the context of nephrotoxicity. The type of antibiotic carrier might affect local release and subsequent systemic absorption, which must be considered.

## 1 Introduction

Infection following orthopaedic trauma surgery remains a major problem. It leads to impaired wound and bone healing, often requiring prolonged hospital stays and multiple revision surgeries. This increases healthcare costs to up to eight times that of non-infected cases (Iliaens et al., 2021; Metsemakers et al., 2017; Haidari et al., 2024). Moreover, despite successful treatment of the condition, final functional and patient reported outcome remains impaired (Walter et al., 2021). As a result, the development of prevention and treatment protocols for fracture-related infection (FRI) has become a critical focus of research in orthopaedic trauma.

The systemic administration of antibiotics holds an integral role in current practice recommendations in both the prevention and the treatment of FRI (Obremskey et al., 2020; Buckman et al., 2022; Depypere et al., 2020). However, despite their widespread use, systemic antibiotics have some important limitations. One major drawback is that the maximal safe concentration, which is determined by toxic side effects, might not be sufficiently high to effectively combat local pathogens at the fracture site. This is particularly relevant in cases where impaired local vascularization further impedes the delivery of systemically administered antibiotics (Metsemakers et al., 2020; Morgenstern et al., 2018).

Local administration serves as a valuable adjunct and achieves high tissue concentrations (Wahlig et al., 1978; Johnson et al., 2017; Janssen et al., 2023). In vitro and in vivo experiments have demonstrated an improved susceptibility to local antibiotics of bacteria that are considered resistant when the antibiotic is administered systemically (Bezstarosti et al., 2024; Metsemakers et al., 2015). Therefore, local antibiotic application of antibiotics has recently gained popularity in the treatment of open fractures and FRI, with multiple studies demonstrating a positive effect of local antibiotic administration in both the prevention and the treatment of FRI (Morgenstern et al., 2018; Flores et al., 2022; Sliepen et al., 2022). Antibiotics can be applied to the fracture site without a carrier (i.e. as a powder), incorporated into a carrier or even adhered to intramedullary nails (Metsemakers et al., 2020; Flores et al., 2022).

Although local antibiotic application offers the advantage of achieving high tissue concentrations while minimizing systemic exposure, systemic absorption cannot be entirely excluded. As aminoglycosides are the most frequently used antibiotic type for local administration, this raises concern for remote organ toxicity (e.g. nephrotoxicity, ototoxicity) (Metsemakers et al., 2020). Aminoglycoside-induced nephrotoxicity is caused by renal tubular toxicity, a reduced glomerular filtration and a reduction in renal blood flow (Wargo and Edwards, 2014). Traditionally, systemic treatment with gentamicin consisted of multiple doses a day, which resulted in nephrotoxicity in up to 25 % of all treatments (Lopez-Novoa et al., 2011; Leehey et al., 1993). Currently, the administration of 5 to 7 mg kg^−1^ body weight once daily is considered equally effective and less nephrotoxic (Wargo and Edwards, 2014). A systematic review and meta-analysis confirmed that trough levels below 2 mg L^−1^ significantly reduce the risk for nephrotoxicity (Yamada et al., 2021). In one additional study, trough levels below 1 mg L^−1^ resulted in a significantly lower incidence of nephrotoxicity compared to through levels above 1.1 mg L^−1^ (Raveh et al., 2002). Future studies are required to assess whether the risk is further reduced at these lower levels (Wargo and Edwards, 2014).

Again, evidence on the safety of local antibiotics mostly stems from PJI-related research. Although most of these studies have shown the local administration of antibiotics to be safe, the occurrence of nephrotoxicity has been described in multiple instances (Luu et al., 2013; Chaudhry et al., 2023; Thomas et al., 2024). In orthopaedic trauma, most studies focus on local antibiotic administration without a carrier (O'Toole et al., 2021; O'Hara et al., 2022). This results in a brief period of high local antibiotic concentrations. The application of an antibiotic carrier is supposed to release the antibiotic over a longer period (Anagnostakos and Meyer, 2017). Data on the safety of this prolonged period of high local antibiotic concentrations are currently lacking.

The aim of this study was to describe the systemic absorption of locally applied antibiotics and the occurrence of nephrotoxicity with current clinical use in orthopaedic trauma surgery.

## 2 Methods

The study was approved by the Medical Research Ethics Committee of the Erasmus MC (MEC-2021-0446) and conducted following good clinical practice guidelines. Patients treated for an open fracture or FRI, diagnosed by the consensus definition (Metsemakers et al., 2018) between 24 November 2021 and 24 June 2024 at Erasmus MC and to whom antibiotics were administered at the fracture site, were eligible for inclusion. Excluded from the study were patients to whom the same antibiotic was administered systemically, adequate follow-up was deemed improbable or no postoperative laboratory values were determined. When eligible for inclusion, patients were approached shortly postoperatively to obtain informed consent.

According to the protocol, postoperative laboratory analyses were routinely performed at 6, 24 and 48 h postoperatively, and subsequently daily until discharge. The plasma gentamicin and vancomycin concentration were analysed with an immuno-assay on an Architect C4000 system (Abbott, Illinois, USA). For gentamicin, the bias and precision of this method were 1.4 % and 4.0 %, respectively. The validated concentration ranged between 0.22 and 10 mg L^−1^. For vancomycin, the bias and precision of this method were -5.1 % and 14.4 %, respectively. The validated concentration ranged between 0.7 and 100 mg L^−1^. During data analysis, measurements were categorized into nine groups: 6 ( ± 6) h, 24 ( ± 6) h, 48 ( ± 6) h, 3 d (72 ± 12 h), 4 d (96 ± 12 h), 5 d (120 ± 12 h), 6 d (144 ± 12 h), 7 d (168 ± 12 h) and later than 1 week ( >180 h). Laboratory analyses included the systemic antibiotic concentration of the locally administered antibiotic, serum creatinine and glomerular filtration rate (GFR). In accordance with the practice guidelines of the Kidney Disease Improving Global Outcomes (KDIGO), a GFR above 90 mL min^−1^ was considered to be normal renal function.

Additional data on patient demographics, the initial trauma, previous treatment, index surgery and perioperative systemic antibiotic administration were collected from electronic patient records. Details on the locally administered antibiotic (type, dose and carrier) were collected from the surgery report or, if not available, from the implant registry. In our hospital, systemic antibiotic prophylaxis in patients with open fractures generally consists of either a cephalosporin or amoxicillin-clavulanic acid for 1 to 3 d, depending on the injury severity. In FRI patients, broad-spectrum antibiotics are started empirically until culture results are determined. If possible, targeted oral antibiotics are started hereafter. Five different types of local antibiotic carriers were used. Palacos R + G^®^ (Heraeus Medical, Wehrheim, Germany) is a poly(methyl methacrylate) (PMMA)-based bone cement containing 500 mg of gentamicin sulfate per 40 mg pack. Copal G + V^®^ (Heraeus Medical, Wehrheim, Germany) is a PMMA-based bone cement containing 500 mg of gentamicin sulfate and 2000 mg of vancomycin hydrochloride per 40 mg pack. Septopal^®^ (Biomet, Warsaw, Indiana, USA) chains consist of PMMA beads containing 7.5 mg (regular) or 2.8 mg (mini) of gentamicin sulfate per bead. Cerament G^®^ (Bonesupport, Lund, Sweden) is an injectable bone graft substitute (BGS), consisting of calcium sulfate, hydroxyapatite and sodium chloride. Every mL of Cerament G delivers 17.5 mg gentamicin sulfate. The Expert Tibial Nail (ETN) PROtect^®^ (DePuy Synthes, Warsaw, Indiana, USA) is coated with a thin layer of poly(D,L-lactic acid) (PDLLA) containing 20–52 mg of gentamicin sulfate depending on the implant size.

Data were collected in Castor Electronic Data Collection and analysed using SPSS for Windows (IBM Corp., Armonk, NY, USA). The normality of continuous data was assessed using the Shapiro–Wilk test. Continuous data are presented as median (P25–P75).

## 3 Results

### 3.1 Patient demographics

During the study period, 52 patients underwent a total of 82 surgeries (range of one to five surgeries per patient). The majority of these patients were male ( n=37 ; 71 %). The median age at inclusion was 47 years (31–59 years). Median BMI was 27 kg m^−2^ (P25–P75: 23–31 kg m^−2^). A minority of the patients received treatment for diabetes ( n=3 ; 6 %) or hypertension ( n=13 ; 25 %) at inclusion. Only seven (9 %) of the surgeries were performed to treat an open fracture. Two of these were performed in polytraumatized patients (ISS > 16). All open fractures involved the lower leg. Two patients had an additional fracture of the ipsilateral femur. Stability was achieved using an intramedullary nail ( n=5 ) or a plate and screw osteosynthesis ( n=2 ). Primary closure was achieved in most cases ( n=5 ). Four patients treated for an open fracture developed an FRI postoperatively and were later included again when treating the infection. In most of the 49 FRI patients, the lower limb was affected ( n=44 ; 90 %). In a third of the FRI surgeries, stability was achieved by plate and screw osteosynthesis ( n=25 ; 33 %). Flap coverage was performed in nine (12 %) of the FRI surgeries.

### 3.2 General laboratory analyses

Gentamicin concentration was measured 272 times after 81 surgeries in 51 patients. Vancomycin concentration was measured 60 times after 21 surgeries in 16 patients. In only one of these, vancomycin measurements were performed without concomitant gentamicin measurements. Four measurements (three for gentamicin and one for vancomycin) were performed outside of the predetermined time windows and excluded. None of the excluded antibiotic concentration measurements was above the lower limit of quantification (LLOQ). Renal function was measured 277 times after 81 surgeries in 51 patients. Of these, seven measurements were excluded because they were performed outside of the predetermined time windows. An overview of the number of included measurements per time point and antibiotic carrier is provided in Table 1.

**Table 1:** Overview of the included number of measurements per time point presented by carrier.

Time point	Spacer			Beads		c-IMN		BGS		Spacer + beads		
	G	V	GFR	G	GFR	G	GFR	G	GFR	G	V	GFR
6 h	32	15	32	20	20	9	9	1	1	4	2	4
24 h	31	14	31	16	17	11	11	1	1	4	3	4
48 h	19	7	18	14	14	8	7	1	1	3	2	4
3 d	15	5	14	10	11	5	5	0	0	3	2	4
4 d	11	3	11	6	6	2	2	0	0	3	1	3
5 d	10	3	10	5	5	2	2	0	0	2	0	2
6 d	6	1	6	3	3	2	2	0	0	2	0	2
7 d	3	1	3	2	2	1	1	0	0	0	0	0
>1 week	0	0	0	0	0	1	1	0	0	1	0	1
Total	127	49	125	76	78	41	40	3	3	22	10	24

BGS: bone graft substitute; c-IMN: coated intramedullary nail; G: gentamicin measurements; GFR: glomerular filtration rate measurements; V: vancomycin measurements.

### 3.3 PMMA spacer

**Figure 1:**
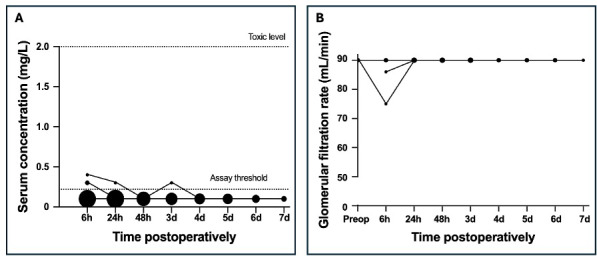
Postoperative serum gentamicin concentration for patients treated with a local PMMA spacer **(A)** and the glomerular filtration rate for the subgroup of patients with gentamicin concentrations above the lower limit of quantification **(B)**. The size of the dots reflects the frequency of occurrence of each concentration, with larger dots indicating more frequent measurements.

A PMMA spacer containing gentamicin or a combination of gentamicin and vancomycin was placed at the fracture site in 19 and 22 surgeries, respectively. The gentamicin dose was unknown in one patient. The median amount of locally applied gentamicin in the other 40 patients was 1000 mg ( $P_{25}$ – $P_{75}$ : 500–1000 mg). The maximum dose was 3000 mg. The vancomycin dose was unknown in two patients. The median amount of vancomycin used in the other 20 surgeries was 2000 mg ( $P_{25}$ – $P_{75}$ : 2000–2000 mg). The maximum dose was 4000 mg. The concentration of gentamicin was above the LLOQ after four (10 %) surgeries (Fig. 1). In three patients, a concentration of 0.3 mg L^−1^ ( n=2 ) or 0.4 mg L^−1^ ( n=1 ) was reached 6 h postoperatively. Only one of these patients (concentration of 0.3 mg L^−1^) showed a brief reduction in renal function (GFR 75 mL min^−1^ at 6 h postoperatively) before a complete recovery at 24 h postoperatively. In the fourth patient, the LLOQ was exceeded once 72 h postoperatively (concentration of 0.3 mg L^−1^) and immediately returned to normal hereafter. The GFR was initially reduced to 86 mL min^−1^ but returned to normal by 24 h postoperatively. None of the patients had a concentration that measured above the LLOQ for vancomycin.

### 3.4 PMMA beads

**Figure 2:**
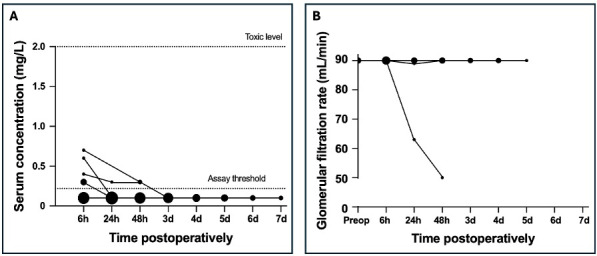
Postoperative serum gentamicin concentration for patients treated with local beads **(A)** and the glomerular filtration rate for the subgroup of patients with gentamicin concentrations above the lower limit of quantification **(B)**. The size of the dots reflects the frequency of occurrence of each concentration, with larger dots indicating more frequent measurements.

As a single carrier of gentamicin, PMMA beads were used during 22 surgeries, performed in 20 patients. The median amount of locally administered antibiotic during these surgeries was 131 mg ( $P_{25}$ – $P_{75}$ : 56–401 mg). The maximum dose was 675 mg. The LLOQ was exceeded after seven (32 %) surgeries (Fig. 2). The highest serum concentrations were measured 6 h postoperatively: 0.3 mg L^−1^ ( n=4 ), 0.4 mg L^−1^ ( n=1 ), 0.6 mg L^−1^ ( n=1 ) and 0.7 mg L^−1^ ( n=1 ). All serum gentamicin levels decreased below the LLOQ by 72 h postoperatively. In one patient (concentration of 0.3 mg L^−1^), the GFR was 89 mL min^−1^ after 24 h but returned to normal after. In another patient (concentration of 0.4 mg L^−1^), the GFR decreased to 50 mL L^−1^ after 48 h without consequential follow-up. In all other patients, renal function remained normal.

### 3.5 Gentamicin coated intramedullary nail

**Figure 3:**
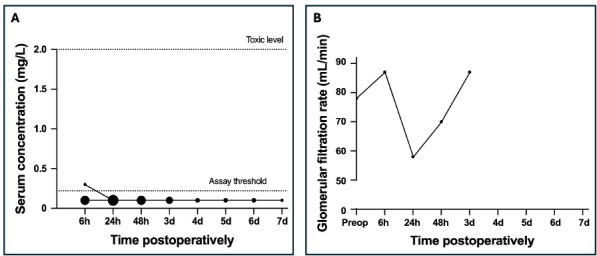
Postoperative serum gentamicin concentration for patients treated with a coated intramedullary nail **(A)** and the glomerular filtration rate for the patient with gentamicin concentrations above the lower limit of quantification **(B)**. The size of the dots reflects the frequency of occurrence of each concentration, with larger dots indicating more frequent measurements.

Coated intramedullary nails were implanted during 12 surgeries. The median gentamicin dose in these patients was 36 mg ( $P_{25}$ – $P_{75}$ : 31–36 mg). The maximum dose was 37.4 mg. The LLOQ was exceeded in only one of these (Fig. 3). In this patient, a serum concentration of 0.3 mg L^−1^ was reached at 6 h postoperatively, which decreased below the LLOQ by 24 h postoperatively. In this patient, the preoperative GFR was 78 mL min^−1^ and fluctuated between 58 and 87 mL min^−1^ before reaching the final measurement of 87 mL min^−1^ at 72 h after surgery.

### 3.6 Combination of antibiotic carriers

During six surgeries, gentamicin PMMA beads were combined with a PMMA spacer containing gentamicin with ( n=5 ) or without ( n=1 ) vancomycin. The median amount of implanted gentamicin and vancomycin was 1002 mg ( $P_{25}$ – $P_{75}$ : 738–1356 mg) and 3000 mg ( $P_{25}$ – $P_{75}$ : 2000–4000 mg), respectively. The LLOQ was exceeded once for gentamicin. In this patient, a gentamicin concentration of 1 mg L^−1^ and a GFR of 82 mL min^−1^ were measured 6 h postoperatively. Preoperative renal function was normal. The patient left the hospital immediately after and no further short-term follow-up was performed. However, the patient was later included again because of another surgery, and their renal function had returned to normal. The LLOQ for vancomycin was never exceeded.

### 3.7 Bone graft substitute

**Figure 4:**
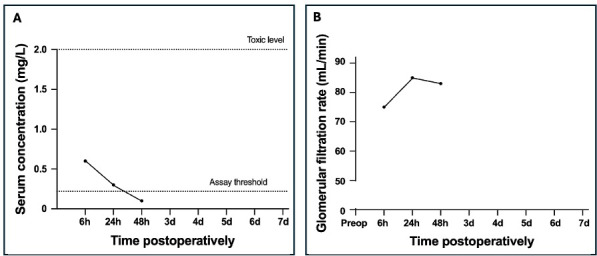
Postoperative serum gentamicin concentration **(A)** and the glomerular filtration rate **(B)** for a patient treated with a synthetic bone graft substitute.

In one FRI patient, a BGS was used to administer 350 mg of gentamicin at the fracture site. A maximum concentration of 0.6 mg L^−1^ was measured after 6 h and returned to below the LLOQ by 48 h postoperatively (Fig. 4). GFR was not measured preoperatively. The GFR was 75 mL min^−1^ at 6 h postoperatively and increased to the final measured value of 83 mL min^−1^ at 48 h postoperatively.

## 4 Discussion

The local antibiotic administration offers a valuable addition in the prevention and treatment of bone infection. As aminoglycosides and vancomycin are the most commonly used antibiotics for local administration, one concern is the potential for absorption into the systemic circulation, which could result in remote organ toxicity (e.g. nephrotoxicity). In this study, data were collected on the systemic antibiotic concentration and occurrence of nephrotoxicity after 82 surgeries performed in 52 patients, during which gentamicin, vancomycin or a combination of gentamicin and vancomycin were administered at the fracture site using multiple carriers to treat an open fracture or FRI.

The vancomycin concentration was determined in 60 samples after 21 surgeries in 16 patients. None of these were above the LLOQ of 0.70 mg L^−1^. These results are in line with recent studies demonstrating the safety of locally administered vancomycin in trauma patients. In a substudy of the VANCO trial, O'Toole et al. (2021) did not find any detectable serum vancomycin levels at 1 h and 6 to 8 h postoperatively in patients treated with 1000 mg of topical vancomycin only. An increase in serum creatinine was found in only one patient with undetectable vancomycin levels. In a follow-up study – a secondary analysis of PREP-IT trial data – O'Hara et al. (2022) found no association between cumulative doses of vancomycin and drug-induced acute kidney injury (AKI).

**Table 2:** Summary of information on locally applied antibiotics and renal function for patients with serum gentamicin levels above the lower limit of quantification.

Surgery	Indication	Antibiotic	Gentamicin	Gentamicin	Preoperative	Lowest	Last	AKI
		carrier	dose (mg)	concentration	GFR	postoperative	measured	
				(mg L^−1^)	(mL min^−1^)	GFR	GFR	
						(mL min^−1^)	(mL min^−1^)	
1	FRI	Spacer	1000	0.3	90	75	90	No
2^*^	FRI	Spacer	1000	0.3	Unknown	86	90	No
3	FRI	Spacer	500	0.3	Unknown	90	90	No
4	FRI	Spacer	3000	0.4	90	90	90	No
5^*^	FRI	Beads	253	0.3	90	90	90	No
6	FRI	Beads	225	0.3	90	90	90	No
7	FRI	Beads	112.5	0.3	Unknown	89	90	No
8	FRI	Beads	56	0.3	Unknown	90	90	No
9	FRI	Beads	112.5	0.4	90	50	50	Yes
10	FRI	Beads	75	0.6	Unknown	90	90	No
11^*^	FRI	Beads	675	0.7	90	90	90	No
12	FRI	c-IMN	31.2	0.3	78	58	87	No
13^*^	FRI	Spacer + beads	1400	1.0	90	82	82	No
14	FRI	BGS	350	0.6	Unknown	75	83	No

AKI: acute kidney injury; BGS: bone graft substitute; c-IMN: coated intramedullary nail; FRI: fracture-related infection; GFR: glomerular filtration rate measurements. ^*^ Two surgeries performed on the same patient.

The gentamicin concentration was determined in 272 samples after 81 surgeries in 51 patients. The LLOQ of 0.22 mg L^−1^ was exceeded only after 14 surgeries in 12 patients (Table 2). All these patients were treated for FRI using different types of antibiotic carriers, with a median gentamicin dose of 302 mg ( $P_{25}$ – $P_{75}$ : 103–1000 mg) per patient. This resulted in a median peak concentration of 0.3 mg L^−1^ ( $P_{25}$ – $P_{75}$ : 0.3–0.6 mg L^−1^). There appeared to be no consistent pattern between the reduction of renal function and either the gentamicin dose or the measured serum concentration. These results are in line with other studies demonstrating that the local application of gentamicin mostly results in serum levels below the toxic threshold of 2.0 mg L^−1^. Maruo et al. (2022) treated 40 patients with FRI by the continuous infusion of gentamicin at a standard dose of 2.4 mg h^−1^ into the medullary cavity or soft tissues, resulting in a constant flow of antibiotics. In all but four patients, the serum concentrations remained beneath the toxic threshold.

Detectable serum levels were found after seven (32 %) of 22 surgeries, in which gentamicin beads were implanted. The concentration range was 0.3–0.7 mg L^−1^. In a previous study, de Klaver et al. (2012) measured serum concentrations after implantation of gentamicin beads during 34 surgeries for infected hip joints and found levels above their LLOQ of 0.40 mg L^−1^ in nine (26 %) of them. The concentrations ranged from 0.40 to 0.97 mg L^−1^. Although all but one patient treated with gentamicin beads resulting in concentrations above the LLOQ received an antibiotic dose below the median, the measured concentrations were above the median concentration more often. With the ratio of total surface area to antibiotic dose in beads being higher than in a single antibiotic spacer, this could indicate a possible effect of this factor on antibiotic release and subsequent absorption in the systemic circulation.

In our study, only one patient was treated with Cerament G^®^ (dose 350 mg). This resulted in a serum concentration of 0.6 mg L^−1^ 6 hours postoperatively, which decreased to below the LLOQ by 48 h postoperatively. In a previous study, Stravinskas et al. (2016) used Cerament G^®^ during the treatment of 19 patients for trochanteric hip fractures, uncemented hip revisions and bone tumour resections, delivering gentamicin doses of up to 350 mg. Similar to our patient, serum concentrations of gentamicin peaked in the first hours postoperatively. However, the levels were frequently higher, occasionally crossing the threshold of 2 mg L^−1^.

Multiple systematic reviews have investigated the association between the implantation of antibiotic-loaded bone cement for the treatment of PJI and the development of AKI (Luu et al., 2013; Chaudhry et al., 2023; Thomas et al., 2024). Although Thomas et al. (2024) describe an incidence of AKI ranging up to 35 %, there is consensus that patient-related factors play a significant contributing role. In our study, preoperative renal function was only assessed when deemed clinically necessary. All but one of these patients had a normal GFR. After half of the surgeries resulting in postoperative measurable gentamicin levels, the GFR was initially reduced (range of 58 to 89 mL min^−1^). The criteria for AKI were met in only one patient. There was no clear correlation between the reduction in renal function and the absolute amount of gentamicin administered or serum concentration measured. The highest measured antibiotic level was 1.0 mg L^−1^, well below the generally accepted toxic trough level of 2.0 mg L^−1^. Like in previous studies, the initial reduction in renal function is therefore more likely attributable to other factors such as the duration and blood loss associated with revision/reconstruction surgery.

This study has several limitations. First of all, measurements of antibiotic concentration are occasionally missed. Based on the available data, concentrations above the LLOQ are mostly expected 6 h postoperatively. With missing measurements at this time point, there is a risk for underestimating the occurrence of antibiotic levels above the LLOQ. Second, the detection limit of our laboratory equipment measuring the antibiotic concentrations prevented measurements of concentrations below 0.22 mg L^−1^ for gentamicin and 0.70 mg L^−1^ for vancomycin. However, this is in line with previous research mentioning detection limits between 0.2 and 0.4 mg L^−1^ (de Klaver et al., 2012; Stravinskas et al., 2016). As our detection limit is still only a 10th of the toxic threshold, the clinical relevance is probably negligible. Third, although no exclusion was performed based on a preoperatively reduced renal function, patients in this study generally had a good preoperative renal function. Therefore, no conclusions can be drawn on the systemic absorption of locally administered antibiotics in patients with a severely reduced renal function. Fourth, when the antibiotic dose was not mentioned in the surgery report, it was collected from the implant registry. Especially with the implantation of an antibiotic spacer, there is a risk of overestimating the antibiotic dose if only part of the cement package was used. Fifth, due to the small number of patients with gentamicin levels above the LLOQ, a detailed assessment of the association between antibiotic dose and serum concentration, or a statistical comparison between the occurrence of AKI in patients with and without gentamicin levels above the LLOQ, were not possible. Lastly, a variety of carriers were used, which has an impact on the local release of the antibiotic. Powders are released much faster, whereas PDLLA elution is longer but slower compared to PMMA. Antibiotic release from PMMA varies not only based on the antibiotic dose but also the type of PMMA, geometry of spacers/beads and mixing method (Nelson et al., 1992; Meyer et al., 2011; Duey et al., 2012; Meeker et al., 2019; von Hertzberg-Boelch et al., 2022).

## 5 Conclusion

This study describes the systemic absorption of locally applied antibiotics and the occurrence of nephrotoxicity with current clinical use in orthopaedic trauma surgery. The results suggest that the current clinical utilization of local antibiotics is safe in the context of nephrotoxicity. However, the type of antibiotic carrier might have an impact on local release and subsequent systemic absorption, which must be taken into account. For spacers and beads, gentamicin doses up to 3000 and 675 mg, respectively, resulted in a serum concentration that was well below the toxic threshold.

## Data Availability

Datasets used during the current study are available from the corresponding author on reasonable request.

## References

[bib1.bib1] Anagnostakos K, Meyer C (2017). Antibiotic Elution from Hip and Knee Acrylic Bone Cement Spacers: A Systematic Review. Biomed Res Int.

[bib1.bib2] Bezstarosti H, Van Lieshout EMM, Van den Hurk MJB, Kortram K, Oprel P, Koch BCP, Croughs PD, Verhofstad MHJ (2024). In Vitro Elution of Gentamicin from CERAMENT(R) G Has an Antimicrobial Effect on Bacteria With Various Levels of Gentamicin Resistance Found in Fracture-related Infection. Clin Orthop Relat Res.

[bib1.bib3] Buckman SA, Forrester JD, Bessoff KE, Parli SE, Evans HL, Huston JM (2022). Surgical Infection Society Guidelines: 2022 Updated Guidelines for Antibiotic Use in Open Extremity Fractures. Surg Infect (Larchmt).

[bib1.bib4] Chaudhry YP, LaGreca M, Hayes H, Papadelis E, Rao SS, Amin R (2023). Acute kidney injury in the context of staged revision arthroplasty and the use of antibiotic-laden cement spacers: a systematic review. J Orthop Surg Res.

[bib1.bib5] de Klaver PA, Hendriks JG, van Onzenoort HA, Schreurs BW, Touw DJ, Derijks LJ (2012). Gentamicin serum concentrations in patients with gentamicin-PMMA beads for infected hip joints: a prospective observational cohort study. Ther Drug Monit.

[bib1.bib6] Depypere M, Kuehl R, Metsemakers WJ, Senneville E, McNally MA, Obremskey WT, Zimmerli W, Atkins BL, Trampuz A, Fracture-Related Infection Consensus Group (2020). Recommendations for Systemic Antimicrobial Therapy in Fracture-Related Infection: A Consensus From an International Expert Group. J Orthop Trauma.

[bib1.bib7] Duey RE, Chong AC, McQueen DA, Womack JL, Song Z, Steinberger TA, Wooley PH (2012). Mechanical properties and elution characteristics of polymethylmethacrylate bone cement impregnated with antibiotics for various surface area and volume constructs. Iowa Orthop J.

[bib1.bib8] Flores MJ, Brown KE, Morshed S, Shearer DW (2022). Evidence for Local Antibiotics in the Prevention of Infection in Orthopaedic Trauma. J Clin Med.

[bib1.bib9] Haidari S, Buijs MAS, Plate JDJ, Zomer JJ, Ijpma FFA, Hietbrink F, Govaert GAM (2024). Costs of fracture-related infection: the impact on direct hospital costs and healthcare utilisation. Eur J Trauma Emerg Surg.

[bib1.bib10] Iliaens J, Onsea J, Hoekstra H, Nijs S, Peetermans WE, Metsemakers WJ (2021). Fracture-related infection in long bone fractures: A comprehensive analysis of the economic impact and influence on quality of life. Injury.

[bib1.bib11] Janssen DMC, Willems P, Geurts J, Arts CJJ (2023). Antibiotic release from PMMA spacers and PMMA beads measured with ELISA: Assessment of in vitro samples and drain fluid samples of patients. J Orthop Res.

[bib1.bib12] Johnson JD, Nessler JM, Horazdovsky RD, Vang S, Thomas AJ, Marston SB (2017). Serum and Wound Vancomycin Levels After Intrawound Administration in Primary Total Joint Arthroplasty. J Arthroplasty.

[bib1.bib13] Leehey DJ, Braun BI, Tholl DA, Chung LS, Gross CA, Roback JA, Lentino JR (1993). Can pharmacokinetic dosing decrease nephrotoxicity associated with aminoglycoside therapy. J Am Soc Nephrol.

[bib1.bib14] Lopez-Novoa JM, Quiros Y, Vicente L, Morales AI, Lopez-Hernandez FJ (2011). New insights into the mechanism of aminoglycoside nephrotoxicity: an integrative point of view. Kidney Int.

[bib1.bib15] Luu A, Syed F, Raman G, Bhalla A, Muldoon E, Hadley S, Smith E, Rao M (2013). Two-stage arthroplasty for prosthetic joint infection: a systematic review of acute kidney injury, systemic toxicity and infection control. J Arthroplasty.

[bib1.bib16] Maruo A, Oda T, Mineo R, Miya H, Muratsu H, Fukui T, Oe K, Kuroda R, Niikura T (2022). Continuous local antibiotic perfusion: A treatment strategy that allows implant retention in fracture-related infections. J Orthop Surg (Hong Kong).

[bib1.bib17] Meeker DG, Cooper KB, Renard RL, Mears SC, Smeltzer MS, Barnes CL (2019). Comparative Study of Antibiotic Elution Profiles From Alternative Formulations of Polymethylmethacrylate Bone Cement. J Arthroplasty.

[bib1.bib18] Metsemakers WJ, Emanuel N, Cohen O, Reichart M, Potapova I, Schmid T, Segal D, Riool M, Kwakman PH, de Boer L, de Breij A, Nibbering PH, Richards RG, Zaat SA, Moriarty TF (2015). A doxycycline-loaded polymer-lipid encapsulation matrix coating for the prevention of implant-related osteomyelitis due to doxycycline-resistant methicillin-resistant Staphylococcus aureus. J Control Release.

[bib1.bib19] Metsemakers WJ, Smeets B, Nijs S, Hoekstra H (2017). Infection after fracture fixation of the tibia: Analysis of healthcare utilization and related costs. Injury.

[bib1.bib20] Metsemakers WJ, Morgenstern M, McNally MA, Moriarty TF, McFadyen I, Scarborough M, Athanasou NA, Ochsner PE, Kuehl R, Raschke M, Borens O, Xie Z, Velkes S, Hungerer S, Kates SL, Zalavras C, Giannoudis PV, Richards RG, Verhofstad MHJ (2018). Fracture-related infection: A consensus on definition from an international expert group. Injury.

[bib1.bib21] Metsemakers WJ, Fragomen AT, Moriarty TF, Morgenstern M, Egol KA, Zalavras C, Obremskey WT, Raschke M, McNally MA, Fracture-Related Infection Consensus Group (2020). Evidence-Based Recommendations for Local Antimicrobial Strategies and Dead Space Management in Fracture-Related Infection. J Orthop Trauma.

[bib1.bib22] Meyer J, Piller G, Spiegel CA, Hetzel S, Squire M (2011). Vacuum-mixing significantly changes antibiotic elution characteristics of commercially available antibiotic-impregnated bone cements. J Bone Joint Surg Am.

[bib1.bib23] Morgenstern M, Vallejo A, McNally MA, Moriarty TF, Ferguson JY, Nijs S, Metsemakers WJ (2018). The effect of local antibiotic prophylaxis when treating open limb fractures: A systematic review and meta-analysis. Bone Joint Res.

[bib1.bib24] Nelson CL, Griffin FM, Harrison BH, Cooper RE (1992). In vitro elution characteristics of commercially and noncommercially prepared antibiotic PMMA beads. Clin Orthop Relat Res.

[bib1.bib25] Obremskey WT, Metsemakers WJ, Schlatterer DR, Tetsworth K, Egol K, Kates S, McNally M, ICM Orthopaedic Trauma Work Group (2020). Musculoskeletal Infection in Orthopaedic Trauma: Assessment of the 2018 International Consensus Meeting on Musculoskeletal Infection. J Bone Joint Surg Am.

[bib1.bib26] O'Hara NN, Carullo J, Joshi M, Banoub M, Claeys KC, Sprague S, Slobogean GP, O'Toole RV, PREP-IT Investigators (2022). Does cumulative topical antibiotic powder use increase the risk of drug induced acute kidney injury in fracture patients?. Bone Jt Open.

[bib1.bib27] O'Toole RV, Degani Y, Carlini AR, Castillo RC, O'Hara NN, Joshi M, METRC (2021). Systemic Absorption and Nephrotoxicity Associated With Topical Vancomycin Powder for Fracture Surgery. J Orthop Trauma.

[bib1.bib28] Raveh D, Kopyt M, Hite Y, Rudensky B, Sonnenblick M, Yinnon AM (2002). Risk factors for nephrotoxicity in elderly patients receiving once-daily aminoglycosides. QJM.

[bib1.bib29] Sliepen J, Corrigan RA, Dudareva M, Wouthuyzen-Bakker M, Rentenaar RJ, Atkins BL, Govaert GAM, McNally MA, Ijpma FFA (2022). Does the Use of Local Antibiotics Affect Clinical Outcome of Patients with Fracture-Related Infection?. Antibiotics (Basel).

[bib1.bib30] Stravinskas M, Horstmann P, Ferguson J, Hettwer W, Nilsson M, Tarasevicius S, Petersen MM, McNally MA, Lidgren L (2016). Pharmacokinetics of gentamicin eluted from a regenerating bone graft substitute: In vitro and clinical release studies. Bone Joint Res.

[bib1.bib31] Thomas TL, Kothari PD, Baker CM, Tarabichi S, Clark SC, Goh GS (2024). High Incidence of Acute Kidney Injury Following Antibiotic-Loaded Spacer Insertion for Periprosthetic Joint Infection: An Updated Review of the Literature. J Arthroplasty.

[bib1.bib32] von Hertzberg-Boelch SP, Luedemann M, Rudert M, Steinert AF (2022). PMMA Bone Cement: Antibiotic Elution and Mechanical Properties in the Context of Clinical Use. Biomedicines.

[bib1.bib33] Wahlig H, Dingeldein E, Bergmann R, Reuss K (1978). The release of gentamicin from polymethylmethacrylate beads. An experimental and pharmacokinetic study. J Bone Joint Surg Br.

[bib1.bib34] Walter N, Rupp M, Hierl K, Pfeifer C, Kerschbaum M, Hinterberger T, Alt V (2021). Long-term patient-related quality of life after fracture-related infections of the long bones. Bone Joint Res.

[bib1.bib35] Wargo KA, Edwards JD (2014). Aminoglycoside-induced nephrotoxicity. J Pharm Pract.

[bib1.bib36] Yamada T, Fujii S, Shigemi A, Takesue Y (2021). A meta-analysis of the target trough concentration of gentamicin and amikacin for reducing the risk of nephrotoxicity. J Infect Chemother.

